# Primary mitral regurgitation, surgery in the transcatheter era: when the neighbourhood becomes noisy: a state-of-art review

**DOI:** 10.1093/ehjimp/qyaf041

**Published:** 2025-04-09

**Authors:** Edoardo Zancanaro, Julia Grapsa, Karl Patrick Kresoja, Guido Ascione, Kabir Sethi, Sebastian Rosch, Davide Carino, Daniel Sebastian Dohle, Michele Di Mauro, Ralph Stephan von Bardeleben, Hendrik Treede, Philipp Lurz, Roberto Lorusso

**Affiliations:** Department of Cardiac Surgery, San Raffaele Scientific Institute, Olgettina Street 69, 20132 Milan, Italy; Cardiovascular Research Institute Maastricht, Universiteitssingel 50, 6229 ER Maastricht, The Netherlands; Department of Cardiology, University Medical Center of the Johannes Gutenberg-University Mainz, Langenbeckstraße 1, 55131 Mainz, Germany; Department of Cardiac Surgery, University Medical Center of the Johannes Gutenberg-University Mainz, Mainz, Germany; Cardiology Department, Guy’s and St Thomas’ NHS Foundation Trust, London, UK; Department of Cardiology, University Medical Center of the Johannes Gutenberg-University Mainz, Langenbeckstraße 1, 55131 Mainz, Germany; Department of Cardiac Surgery, San Raffaele Scientific Institute, Olgettina Street 69, 20132 Milan, Italy; Department of Cardiology, University Medical Center of the Johannes Gutenberg-University Mainz, Langenbeckstraße 1, 55131 Mainz, Germany; Delhi Heart and Lung Institute, Panchkuian Road, Metro Station R.k. Ashram Marg, 2, Panchkuian Marg, near RK, Type 4, Block B, Aram Bagh, Paharganj, New Delhi, Delhi 110055, India; Department of Cardiology, University Medical Center of the Johannes Gutenberg-University Mainz, Langenbeckstraße 1, 55131 Mainz, Germany; Cardiac Surgery Unit, Department of Medicine and Surgery, Parma University, Azienda Ospedaliero Universitaria di Parma, Parma, Italy; Department of Cardiac Surgery, University Medical Center of the Johannes Gutenberg-University Mainz, Mainz, Germany; Cardiovascular Research Institute Maastricht, Universiteitssingel 50, 6229 ER Maastricht, The Netherlands; Department of Cardiology, University Medical Center of the Johannes Gutenberg-University Mainz, Langenbeckstraße 1, 55131 Mainz, Germany; Department of Cardiac Surgery, University Medical Center of the Johannes Gutenberg-University Mainz, Mainz, Germany; Department of Cardiology, University Medical Center of the Johannes Gutenberg-University Mainz, Langenbeckstraße 1, 55131 Mainz, Germany; Cardiovascular Research Institute Maastricht, Universiteitssingel 50, 6229 ER Maastricht, The Netherlands; Cardio-Thoracic Surgery Department, Heart & Vascular Centre, Maastricht University Medical Centre, Maastricht, The Netherlands

**Keywords:** primary mitral regurgitation, TEER, repair, state of art review

## Abstract

Primary mitral regurgitation is a valvular heart disorder that found to be treated with surgery that has been the gold standard for decades with different techniques to approach this pathology. In the last decade, Transcatheter Edge-to-Edge Repair emerged as a valid option for this type of pathology, in case of surgical unsuitability. Others device emerged as well leaving less remark compared with Transcatheter Edge-to-Edge Repair. The article analyse the old and new frontiers as well as the controversies of this phenotype.

## Graphical Abstract

**Graphical Abstract:**
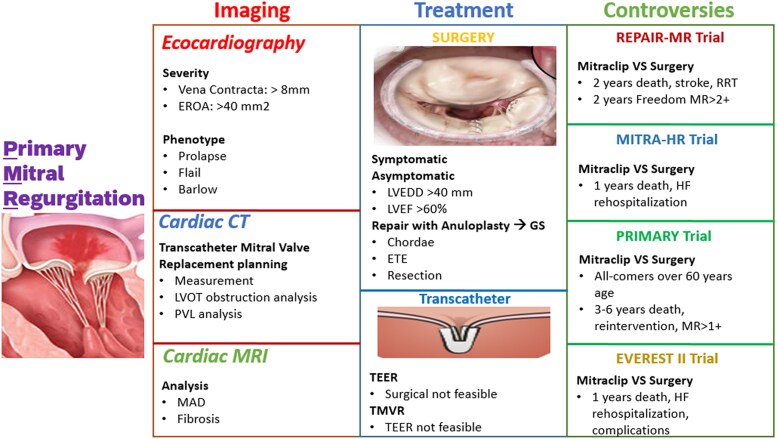

## Introduction

Primary mitral regurgitation (PMR) is a valvular heart disorder in which intrinsic abnormalities of the mitral valve (MV) apparatus lead to backward blood flow from the left ventricle into the left atrium during systole.^1,2^ These abnormalities can result from degenerative changes in the valve leaflets, myxomatous infiltration, calcification of the annulus, or damage to the chordae tendineae.^1^ Unlike secondary mitral regurgitation (MR), which arises due to remodelling of the left ventricle, primary MR originates directly from the lesions of the MV or its supporting structures.^3^ Many patients remain asymptomatic for extended periods, yet progressive left atrial and ventricular enlargement may occur over time, increasing the risk of atrial fibrillation and eventual heart failure.^4^ When symptoms emerge, they commonly include fatigue, exertional dyspnoea, and palpitations.

Surgery has been the gold standard for decades with different techniques to approach this pathology. In the last decade, Transcatheter Edge-to-Edge Repair (TEER) emerged as a valid option for this type of pathology, in case of surgical unsuitability. Other device emerged as well leaving less remark compared with TEER.

The current state-of-art review aimed to elucidate the role of surgery in PMR where transcatheter approach has taken lots of the patients’ panorama but also refresh the pitfalls in this more debated pathology.

## Pathophysiology

PMR arises from intrinsic abnormalities of the MV apparatus. In degenerative MV disease—often cited as the most prevalent aetiology of primary MR—myxomatous changes and fibro-elastic deficiency weaken and elongate leaflets and chordae tendineae, ultimately causing leaflet prolapse or flail segments.^3^ Such disruptions prevent normal leaflet coaptation, forming a regurgitant orifice and imposing volume overload on the left atrium (LA). In response, the LA undergoes compensatory dilation and remodelling to handle the increased volume; initially, this adaptation keeps left atrial pressures relatively low, allowing many patients to remain asymptomatic for extensive periods.^4^ However, as the regurgitant volume (RVol) grows, the ability of the LA to accommodate the overload diminishes, causing elevations in left atrial pressure and symptom onset, typically exertional dyspnoea, palpitations, and a reduced exercise capacity.

Meanwhile, the left ventricular (LV) faces both forward and backward stroke volumes, leading to an increased end-diastolic volume and prompting an eccentric pattern of ventricular hypertrophy.^4^ During the early stages, these compensatory changes may sustain adequate stroke volume and cardiac output. Over time, prolonged volume overload can degrade LV function, manifesting through rising end-systolic volume and a drop in ejection fraction.^3^ Persistent strain on the LA can also predispose patients to atrial fibrillation, further exacerbating haemodynamic compromise by abolishing synchronized atrioventricular contraction.^4^ This interplay of elevated left atrial pressures, potential arrhythmias, and progressive LV remodelling not only undermines overall cardiac performance but also fosters a cascade leading to clinical heart failure.

## Multimodality imaging

### Transthoracic echocardiography

Echocardiography is determinant for appropriate patient selection and successful outcomes. In MR, echocardiographic assessment is divided into pre-, intra-, and post-procedural. As a first step pre-procedural, it is important to assess the anatomy of the MV and especially all scallops and commissures, if appropriate to classify the aetiology according to the Carpentier classification.^5^ An important part of pre-procedural assessment is to detect indentations vs. clefts as in the scenario of a congenital cleft, specific intervention such as the insertion of MitraClip may not be applicable. Important enough is the assessment of the mitral annular calcification and the assessment of the mitral annulus. The latter is often described as having a saddle-shaped morphology on 3D studies with anterior and posterior peaks, and with nadirs near the fibrous trigones.^6,7^ Mitral annular disjunction is also important to be identified and if present then cross-sectional imaging with computed magnetic resonance (CMR) may be useful to exclude LV fibrosis.^8^

An important parameter in the assessment of aetiology of MR, is the assessment of LV systolic function as well as right ventricular cavities and the possible co-existence of significant tricuspid regurgitation.^9^ LV global longitudinal strain (GLS) is important as improvement in LV GLS at 6-month follow-up was associated with improved outcomes after both transcatheter edge-to-edge repair and guideline directed medical therapy alone between 6 and 24 months.^10^

MR is being assessed with semi-quantitative methods.^5–10^ Due to the haemodynamic influence of the regurgitant jet with the increase of left atrial pressure and small area jet, colour flow area should be avoided for MR severity quantification.

- With regards to the flow convergence zone with continuous wave signal, severe MR is associated with a large central jet (usually >8 cm^2^ or >50% of left atrial area) or an eccentric jet swirling and reaching the posterior wall of the left atrium (Coanda effect) Flow convergence is not always hemispheric but rather an ellipsoid shell. Severe MR provides a large convergence zone throughout systole.
- Vena contracta (VC) width is highly dependent on the orifice and it is circular in primary MR; non-circular in secondary MR (elongated along the mitral coaptation line). In the scenario of multiple MR jets, the respective widths of the multiple VC widths are not additive. In 2D echo, a VC (biplane) ≥8 mm is severe while in 3D cross-sectional area, severe MR is when VC is ≥40 mm^2^.
- Effective orifice area (EROA) and RVol are best estimated with 3D echocardiography. Severe MR indicates an EROA ≥ 40 mm^2^ and RVol ≥ 60 mL.
- Pulmonary venous flow systolic reversal is best evaluated from the right upper pulmonary vein on transthoracic echocardiography but gold standard is the transesophageal echocardiogram (TOE) assessment with pulsed wave Doppler with the sample placed ∼1 cm deep into the pulmonary vein. Atrial fibrillation and elevated left atrial pressure can blunt forward systolic flow.
- A dense MR signal with continuous wave (CW) with a full envelope indicates severe MR. The envelope may be truncated with a triangular contour and an early peak velocity. In eccentric MR, it may be difficult to record the full CW envelope of the jet.
- Anterograde velocity of mitral inflow—mitral to aortic velocity time integral (VTI) ratio: higher flow velocities during early diastolic filling (increased E velocity). In the absence of mitral stenosis (MS), a peak *E* velocity >1.2 m/s suggests severe MR.
- Dominant A wave (atrial contraction) basically excludes severe MR while a VTI ratio >1.4 suggests severe MR.

### Transoesophageal echocardiography

TOE allows assessment of scallops and indentations and it is fundamental in the guidance of transcatheter procedures. Mid-oesophageal commissural (50–70) and long axis (120–140) views are key to image the mitral leaflet coaptation zone. Commissural view should aim to visualize the symmetric display of papillary muscle heads and chords. TOE will guide the device into the chordal free zone that is why coaxiality is very important. In order to optimize coaxiality, the key is to withdraw the probe and/or using anteflexion will bring the lateral commissure into view, while advancing the probe and/or further retroflexion focuses on the medial structures. Clockwise and counter clockwise rotation of the transesophageal echocardiogram (TEE) probe moves the imaging plane anteriorly and posteriorly.

Mitral anatomical complexity was defined in the EXPAND registry^11,12^ by the following criteria: wide coaptation gap (≥15 mm), large flail gap (≥10 mm), jet outside anterior 2/posterior 2 (A2/P2), small mitral valve area (MVA), calcified landing zone, and minimal leaflet tissue. Patients meeting one or several of these criteria were more likely to achieve procedural success as defined by residual MR ≤ 1+ following M-TEER using the MitraClip device. Furthermore, the presence of annular and leaflet calcifications, in particular leaflet infiltration of 6 mm or more, MVA < 4 cm^2^, baseline trans-mitral gradient ≥4 mmHg, and multiple jets have been identified as risk predictors for an increased final trans-mitral gradient (≥5 mmHg) after M-TEER.^11^

In those patients with significant mitral annular calcifications (MAC), prosthetic valves or devices, the quality of TEE may be challenging and therefore 3D intracardiac echocardiography may have a rising value in these patients.

Understanding the anatomy, aetiology, and echocardiographic features of MR is very important steps in classifying the suitability for edge to edge repair or replacement with more advanced devices, *Table 1*.

**Table 1:** Technical summary on the anatomical indication for TEER

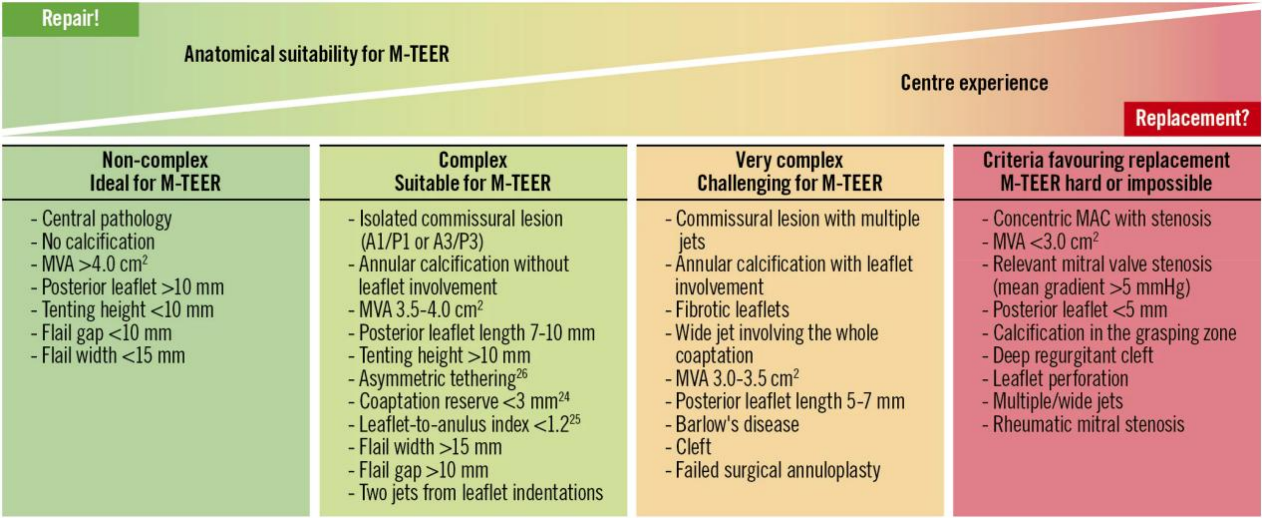

## The value of multiplanar reconstruction

Simultaneous visualization of the MV in three different axis planes (commissural, long axis, and short axis) at the same time is being achieved via multiplanar reconstruction (MPR). MPR allows more accurate localization of the leaflet pathology and resultant regurgitant jet to target during the procedure. 3D MPR allows a detailed assessment of the MV orifice area and annular shape, calcification, and function, which are extremely important in predicting the risk of MV stenosis.

### Cardiac computed tomography

As transcatheter MV replacement has evolved, there is more need of accurate annular sizing and valve simulation to predict complications such as neo-left ventricle outflow tract obstruction and paravalvular leak. More so than any other modality, cardiac computed tomography (CT) remains instrumental in accurately planning transcatheter mitral valve replacement (TMVR) from feasibility, device sizing, access, and fluoroscopic angles. Cardiac CT remains the key modality in TMVR evaluation, often the first step in determining patient eligibility through comprehensive procedural planning as well as informing potential outcomes and prognosis. In this review, we discuss the critical role of cardiac CT and the specific considerations involved in TMVR.^13^

### Cardiac MRI

CMR has an important role in MR and mitral annular disjunction in the detection of fibrosis and risk stratification of patients with arrhythmogenic MV prolapse.^14^ CMR 4D flow has an emerging role in the assessment of challenging MR jets and accurate quantification. Furthermore, CMR will provide a lot of information on scarring and viability in those patients with concomitant coronary artery disease.^15^

## How to assess PMR for intervention

Recognizing the underlying mechanism of MR and grading its severity is of utmost importance in evaluating patients with degenerative or organic MR as it can have implications with respect to patient treatment and prognosis.

As discussed earlier, there are a number of ways in which a patient with organic MR can present. Pathologically, organic MR can result either from a fibro-elastic deficiency or from myxomatous degeneration^16–18^ and encompasses a variety of lesions (billowing, prolapse, and/or flail) which can be differentiated from one another based on simple characteristics on echocardiogram specific to these lesions. In simple words, billowing refers to increased curvature of the leaflets whereas prolapse refers to shifting of the coaptation point above the annular plane. Pathological Billowing is also referred to as prolapse. However a true prolapse has certain distinct characteristics, viz. the distance between the body of the leaflets and the annular plane is more than 2 mm in the long axis view and more than 5 mm in the apical four chamber view and the tip of the leaflet is always directed towards the LV apex. This is of prime importance as it aids in differentiating a prolapse from a flail leaflet (discussed subsequently) where the tip of the involved leaflet is directed towards the left atrium. However, at times a prolapsed leaflet may not be appreciated in its entirety on 2D echo and may be misdiagnosed as a flail such that the observed distal part of the prolapsed segment seems to be directed towards the left atrium, whereas the tip is actually displaced towards the apex.

A flail leaflet usually results from eversion due to ruptured chordate. Its presence is highly supportive of severe MR and suggestive of poor outcome.^19^ For diagnosing a flail leaflet using 2D echocardiography, in addition to the ruptured chordae, there must also be clear visualization of the rapid systolic movement of the tip of the involved leaflet towards the left atrium.

Myxomatous degeneration of the MV is characterized by thickening of the leaflets due to excess tissue and is often found associated with elongated chordae. Diffuse and generalized myxomatous degeneration leads to Barlows disease which involves multiple segments of the leaflets, usually accompanied by a large annulus, multi scallop or a bileaflet prolapse/billowing, thickened or spoongy leaflets (due to excessive myxomatous proliferation) with or without calcification, and reduced protosystolic contraction due to increase in the intercommissural distance.

While evaluating a patient with Barlows, one must always look for the presence of mitral annular disjunction which appears as a clear separation between the mitral annulus (posterior aspect) and the inferolateral wall of the LV myocardium (measured at the end of the systole), and has the potential to cause life threatening ventricular arrhythmias.^20^

Rheumatic heart disease affecting the MV is characterised by variable leaflet thickening (primarily involving the free margins of the leaflets), commissural fusion, and restricted movements of the leaflets in both systole and diastole. In addition, one can also appreciate thickening and fibrosis of the chordae tendinae (and the sub-valvular apparatus) which can lead to two kinds of rheumatic MR, the first, in which the chordae afferent to both the leaflets are involved and the second, in which the chordae afferent only to the posterior leaflet are involved, leading to a pseudo prolapse and a somewhat eccentric MR.^21^Symptomatic patients with severe MR usually receive immediate attention owing to the morbidity and disabling symptoms. On the other hand, asymptomatic individuals usually go unnoticed. Regardless of symptoms, if a patient with severe MR exhibits LVEF < 60%, left ventricle end systolic diameter (LVESD) > 40 mm or a progressive decline in LV function (even if it is more than 60%) or a progressive increase in the LVESD (even if it is lower than 40 mm) on serial echocardiograms, he or she must be considered for early surgery^9^ as early intervention in these patients can mitigate the risk of new onset atrial fibrillation, progressive decline in LV function, and pulmonary artery hypertension. Some of the other parameters which indicate early surgery include resting pulmonary artery systolic pressure > 50 mmHg, LA diameter >55 mm, LA volume >60 mL/m^2^, and the presence of atrial fibrillation.^9^

Those patients who are deemed inoperable in view of prohibitive surgical risk or extreme frailty may be considered for percutaneous edge to edge repair provided the valve is rendered suitable for M-TEER.^5^ Flail width, flail gap, MVA and gradient at baseline, calcium in the grasping zone, posterior leaflet length, and mobility in the grasping zone are some of the parameters which help to assess the suitability of the procedure.

## Treatment

### Surgery

Surgical repair can provide the best early and late outcomes in the setting of degenerative MR. In referral centres, valve repair is feasible in almost all patients.^22^ A timely operation performed before the onset of symptoms or left ventricle dilation or dysfunction can leave the patient with a competent valve which can ideally translates to a survival expectancy similar to that of the general population.^23^ According to current European^9^ and US guidelines, in PMR, MV repair is indicated in patients with symptoms and/or LV dysfunction. In asymptomatic patients with PMR and preserved LV function, surgery should be considered if atrial fibrillation or pulmonary hypertension is present. Surgery should also be considered in asymptomatic patients with PMR who are in sinus rhythm with preserved LV ejection fraction (≥60%) and LV end-systolic diameter >40 mm according to the US guidelines, and 40–44 mm for the European guidelines, when a durable MV repair is likely, surgical risk is low and the MV repair is performed in a heart valve centre.

In degenerative MR, the traditional surgical access through a full median sternotomy has been accompanied by new minimally invasive approaches such as right minithoracotomy, partial sternotomy or a totally endoscopic approach, with or without robotic assistance.

In degenerative MR, prolapse lesions are often ranging from a single scallop involvement (most commonly P2) to multi-segment prolapse (Barlow disease).^24^

Regarding the surgical techniques, ideally a MV surgeon should master the different repair techniques and apply them according to the surgical findings. Regardless the technique employed, surgical MV repair in degenerative disease is always completed with an annuloplasty.

Posterior leaflet prolapse can be treated using resection and non-resection techniques.^25^ Triangular or quadrangular resection can be used, often in association with folding or sliding plasty to effectively decrease the height of the posterior leaflet and decrease the risk of post-operative systolic anterior motion (SAM). A non-resection strategy by means of artificial chordae implantation is also frequently adopted in case of single scallop involvement. No clear differences in results have been observed between these two approaches.^26^ The choice of the technique to be employed relay to the operating surgeon according to his own expertise and to the characteristic of the lesion. As a general rule in the case of pure fibro-elastic deficiency with flail and very thin leaflets, a non-resection approach is usually preferred due to the higher risk of sub-optimal repair using a resection technique due to the frailty of the leaflets. While in case of single scallop prolapse due to myxomatous disease and excessive tissue in the prolapsing scallop both resection and non-resection technique can be safely applied.

Degenerative MV disease affecting exclusively the anterior mitral leaflet is less frequent than posterior or bileaflet disease, and multiple large series have suggested compromised durability after MV repair for this entity.^27^ From an anatomical perspective, the anterior leaflet is characterized by a lesser degree of redundancy compared with the posterior leaflet despite its larger surface area. The number of chordae is lower compared with the posterior leaflet, and most of them are concentrated on the leading edge of the leaflet itself. In addition, investigators have observed differences in the forces applied to the leaflets during LV contraction which have been proposed to account for the reported inferior durability of anterior leaflet defect repair compared with that of the posterior leaflet. For all these reasons repair of anterior leaflet prolapse is more complex than repair of posterior leaflet prolapse Various techniques can be used, including, neo-chordae implantation, edge-to-edge technique, chordal transfer or transposition, and papillary muscle repositioning.^28^

Finally, in case of single scallop involvement with commissural lesions several surgical techniques have been proposed, including papillary muscle repositioning, leaflets’ resections with sliding, isolated neo-chordae implantation and chordal transposition and commissural closure.^29^

A combination of the technique listed is usually necessary to treat bileaflet prolapse, which is typically encountered in the context of Barlow disease. However, several case of bileaflet prolapse can be efficiently treated with an isolated edge-to-edge between A2 and P2. The rational of the edge-to-edge technique in bileaflet prolapse is that a properly placed stitch that encompasses a large amount of leaflet tissue is effective in shortening the height of both leaflets and lowering the coaptation point to within the left ventricle and thus eliminating the regurgitation and avoiding the risk of post-operative SAM.^30^

As anticipated, annuloplasty has a very important role in the repair of PMR and is always used to complete the valve repair using complete ring and posterior band. Also in this situation no clear differences in results have been observed between these two types of annuloplasty.^31^

The durability of MV repair is related to the mechanism of MR, to the absence of residual regurgitation at the end of the procedure and to experience of the centre. The best long-term results of MV repair have been obtained in patients with isolated prolapse of the posterior leaflet. Less favourable outcomes have been reported in patients with MR owing to anterior or bileaflet prolapse. Leaflet erosion and annular calcification are two of the conditions that markedly decrease the likelihood of successful repair, and such patients should be referred to experienced centres with a multidisciplinary heart team to maximize the likelihood of a durable repair or should undergo to MV replacement.

### Transcatheter and medical therapy

There is no data that support the use of medical therapy in patients with chronic PMR, in the absence of left-sided heart failure (HF).^9^ In some patients with chronic PMR transition to concomitant left-sided HF may occur and corresponding guideline directed medical therapy is recommended. Two substance classes that have shown an effective therapeutic profile over a range of LV ejection fraction and left-sided HF—SGLT-2 inhibitors and mineralocorticoid receptor antagonists—are likely suitable early therapeutic choices. In patients with acute PMR, medical and mechanical reduction of filling pressures, is a key therapeutic strategy for early stabilization and to allow for definitive establishment of surgical or transcatheter therapy.^9^

Based on the data of the EVEREST II trial, a trial that compared surgery with M-TEER in patients with both PMR and secondary mitral regurgitation (SMR), current European Society of Cardiology guidelines recommend surgical treatment over M-TEER in patients with PMR at suitable surgical risk.^32^This is due to the fact that in a not pre-specified subgroup analysis patients with PMR showed more favourable results when randomized to surgery as compared with M-TEER. However, M-TEER was at an early stage of its development and only first generation MitraClip devices were available, with only 43% of patients showing a mild or less MR grade in the M-TEER arm as compared with 76% of patients in the surgical arm.^32^ Since then, interventionalists and imagers have seen a vast increase in their understanding and expertise of M-TEER and the industry has increased the armamentarium of M-TEER devices to never seen numbers. Modern M-TEER devices improved clip deployment sequences and have introduced independent leaflet grasping, and different devices sizes to tailor treatment for PMR patients. In line with this the prospective EXPAND G4 registry showed real-world data where by using fourth generation MitraClip devices MR can be reduced to mild or less in 88.4% of patients,^33^ even surpassing surgical results seen in the EVERST II trial^32^ (*P* < 0.001). In patients with prohibitive surgical risk the PASCAL device has proved its non-inferiority in the CLASP-IID trial, with regards to MR reduction, as compared with the MitraClip device (77.1% for PASCAL and 71.3% for MitraClip),^34^ albeit overall MR reduction to mild or less was lower as compared with reports from the EXPAND G4 study.^33^ Improvements in M-TEER devices have even made complex anatomies of PMR treatable, with also significant improvements of MR grades at 1-year ranging from 58 to 86%.^35,36^

With regards to mortality contemporary cohorts have shown mortality rates between 8.6 and 12.3% which are higher as compared with the 6% reported for both surgically as well as percutaneously treated patients from the EVEREST II trial.^33,34^However, this has to be seen against the background of the EVEREST II trial recruiting surgically suitable patients for MV repair, while present M-TEER PMR studies only recruited patients with high or prohibitive surgical risks. In comparison the EVEREST II HRR and REALISM HR studies, which included comparable patients treated with first generation MitraClip devices reported 1-year mortality rates of 22.8%.

Currently there are no data that compare contemporary percutaneous treatment approaches to surgical PMR repair or replacement, leaving physicians with a gap in knowledge on how to optimally treat those patients. Approximately half of the patients presenting with MR are considered inoperable or at high operative surgical risk, and if anatomically suitable modern M-TEER is a safe and effective treatment option for these patients. For patients that are suitable for surgery the situation is more complex. The recently published MATTERHORN trial which investigated M-TEER vs. surgical repair or replacement in patients with SMR might shed some further insights in the present absence of data.^37^ In this trial M-TEER was shown to be non-inferior to surgery with regards to a composite endpoint of death, hospitalization for heart failure, mitral valve reintervention, implantation of an assist device, or stroke within 1 year after the procedure (16.7 vs. 22.5%, *P* < 0.001 for non-inferiority). The M-TEER approach was further associated with a significantly lower rate of adverse events like bleeding complications but also a higher incidence of de novo onset of atrial fibrillation (3.1 vs. 27.8%) as compared with surgery. However, while these data encourage a high safety and efficacy profile for M-TEER as compared with surgery in operable patients, these data are only valid for SMR patients. Accordingly two studies have set out to investigate on the efficacy of M-TEER in patients with high (MITRA-HR, NCT0327176)^38^ and intermediate surgical risk (REPAIR-MR, NCT04198870)^39,40^ in comparison to surgery.

In conclusion, new innovations and developments have made M-TEER both an effective and safe therapeutic device for patients with PMR at prohibitive surgical risk. Whether those favourable results can be translated to patient populations with lower surgical risk, will be determined in future trials that will hopefully expand the therapeutic options that we might offer patients tomorrow.

## Controversies: trials vs. registries and real-world experience

Both European and American guidelines suggest surgical repair as the mainstay of treatment for degenerative MR.^9^ In fact, multiple studies have demonstrated the superiority of repair over replacement in terms of long-term mortality, with the first approach being able, when performed timely, to restore patients’ normal life expectancy.^9^

In this context, transcatheter edge-to-edge repair (TEER) has emerged as an alternative option to treat high-risk, surgical ineligible patients, and whether to extend its indication also to lower risk cohorts is still a matter of debate.

First, in the absence of reliable transcatheter annuloplasty devices and with transcatheter MV replacement still far from becoming a solid therapeutic option, surgical MV repair still remains the only treatment able to address both components of degenerative MR: structural leaflets abnormalities and annular dilatation. The importance of tackling these issues simultaneously has been indeed confirmed by the unsatisfactory long-term results of surgical edge to edge repair without annuloplasty, with a freedom from more than moderate MR of only 43 ± 7.6% at 12 years.

Secondly, many degenerative MR patients have Barlow’s disease (with multiple regurgitant jets, deep clefts, and commissural prolapses) or MAC, both representing yellow or red zone anatomy for TEER, i.e. challenging situations in which an optimal result (mild or less MR at discharge, no significant post-repair gradient) is hard to achieve.^9^

The only randomized-controlled evidence available to date on the topic comes from the EVEREST II trial. In this investigation, who randomized 279 subjects with significant primary or secondary MR to undergo either TEER or MV surgery and finally compared 184 TEER patients with 95 surgical ones (14% MV replacement, 86% MV repair), the primary composite endpoint of freedom from death, mitral reinterventions, and more than moderate MR at 12 months occurred in 55% of the percutaneous-repair group and 73% of the surgery group (*P* = 0.007). Moreover, a subgroup analysis showed that surgery outperformed TEER especially in patients under 70 years of age, with preserved ejection fraction or degenerative MR (the latter category comprising about 70% of both cohorts).^32^

However, the results of this trial are quite historical. Indeed, it enrolled patients between September 2005 and November 2008 and, since then, surgical outcomes have improved (also thanks to a widespread diffusion of minimally invasive techniques) and TEER technology has rapidly evolved, with the introduction of new generation devices [i.e. Pascal (Edwards Lifesciences, Irvine, USA) and Mitraclip G4 (Abbott, Santa Clara, USA)] conceived to overcome the anatomical limitations of the previous ones. Through longer arms and multiple sizes, these new tools are designed to optimize grasping also in patients with very redundant leaflets or commissural lesions, eventually leading to some reduction of annular diameters.

The EXPAND G4 registry reported the 1-year outcomes of the current fourth generation Mitraclip devices. In primary MR patients, mild or less MR was achieved in 90.1% of the patients at 30 days and 88.8% at 1 year. Moreover, in patients with complex MV anatomy (including MAC and Barlow’s disease), 1-year freedom from more than mild MR was still 90.3%.

Conversely, a real-world analysis of 19 088 all-comers primary MR subjects from Transcatheter Valve Therapies registry showed that, at 30 days, an optimal result (mild or less MR and a mean mitral gradient ≤5 mmHg) was achieved in only 52.4% of the cases, while 36.6% of the patients were discharged with either moderate MR or moderate MS and 11% with either severe MR or severe MS. Moreover, after TEER failure, a subsequent surgical repair appears unlikely. In fact, although mitral repair after TEER is technically feasible, real-world registries taught us that up to 95% of patients ultimately undergo MV replacement in case of MR recurrence after percutaneous repair.

In this context, various single-centre surgical series have set the benchmark for successful treatment of primary MR, showing how, especially if performed in Centres of Excellence, MV repair is associated with remarkable short- and long-term outcomes (freedom from more than moderate MR up to 98% at 10 years). The UK Mini Mitral trial (a multicentre randomized trial comparing a minimally invasive-thoracoscopically guided right minithoracotomy vs. conventional sternotomy for MV repair) further confirmed this excellent single-centre results, with a reported 1-year freedom from more than mild MR of 99.9% irrespective of the surgical approach (vs. 76% in the surgical arm of the EVEREST II trial).

Despite the conflicting evidence, the absence of proper comparative results on percutaneous repair vs. surgery in this context and the lack of long-term data on TEER durability, TEER is increasingly used in degenerative MR across the world. Chikwe *et al.*^41^ demonstrated (using insurance-linked data on 53117 MV interventions) that, since 2019, TEER has been performed more frequently than surgical repair in the USA in degenerative MR patients older than 65 years. This is consistent with German national data, which reported 6353 isolated surgical MV procedures and 7434 catheter-based ones in 2022.^41^

There is, therefore, an urgent need of randomized clinical trials (RCT) comparing the outcomes of TEER and surgery in patients with primary MR, with a focus on lower risk cohorts.

There are three ongoing investigations on the topic.

The MITRA-HR trial (NCT03271762) is an investigator initiated, French RCT, enrolling high-risk degenerative MR patients. The composite primary endpoint includes all-cause mortality, unplanned rehospitalisation for cardiovascular reasons, and MV re-intervention at 12 months. The sample size is 330 subjects, with the first one enrolled in 2018.

REPAIR-MR (NCT04198870) is an Abbott sponsored RCT, focusing on moderate-risk subjects. The goal of this trial is to enrol 500 surgical-eligible patients and randomize them to either surgical repair or TEER with Mitraclip. The study has two co-primary endpoints: the composite of 2-year death, stroke, heart failure hospitalizations or need of renal replacement therapy and the proportion of patients with moderate-or-less MR without MV re-interventions at 2 years. The first participant was enrolled in 2020.

Lastly, the PRIMARY trial (NCT05051033) is a National Institute of Health (NIH) founded study, enrolling a population of all-comers, older than 60 years primary MR subjects. The primary effectiveness endpoint is a 3- and 6-year composite of all-cause mortality, any mitral re-intervention, heart failure hospitalizations, or more than mild MR. The primary safety endpoint is the composite of stroke and major bleeding. This trial is rapidly enrolling his sample size of 450 patients.

A comparison of these three trials with the historical EVEREST II trial is provided in *Table 2*.

**Table 2:** RCT comparing TEER and surgery in primary MR patients

	EVEREST II	MITRA-HR	REPAIR-MR	PRIMARY
Sponsor	Abbott Vascular, USA	Nantes University Hospital, France	Abbott Vascular, USA	National Institutes of Health, USA
Trial design	Non-inferiority	Non-inferiority	Non-inferiority	Superiority
Enrolled population	All-comers MR patients (70% primary MR, 30% functional MR)	High-risk patients with primary MR	Moderate-risk patients with primary MR	All-comers, older than 60 years of age patients with primary MR
Treatment arms	Mitraclip vs. Surgery	Mitraclip vs. Surgery	Mitraclip vs. Surgery	TEER vs. Surgery
Primary endpoint	1-year death, MV re-intervention, MR > 3+ Death, MV re-intervention, MI, stroke, AKI, infection, prolonged ventilation, AF, transfusion of >2 blood units	1-year death, cardiovascular re-hospitalization, MV re-intervention	2-year death, stroke, HFH, need of renal replacement therapy 2-year freedom from MR > 2+ without MV re-interventions	3- and 6-year death, MV re-intervention, HFH, MR > 1+ Freedom from stroke and major bleeding
Sample size	272	330	500	450

The results of these ongoing studies, which will be available in the near future, will help the cardiovascular community better select which patients are expected to benefit the most from each treatment option. AKI: acute kidney injury. AF: atrial fibrillation, HFH: heart failure hospitalization.

## Lead author biography

Edoardo Zancanaro is a cardiac surgery resident who graduated in International Medical Program at Università Vita Salute San Raffaele in Milan, Italy, and he is currently resident at San Raffaele Hospital in Milan. He is also performing an advanced fellowship in minimally invasive cardiac surgery and Interventional Cardiology at University Hospital in Mainz.

The principal goal is to become a hybrid cardiac surgeon with a specific attention to valvular heart diseases. The major topics of interest are minimally invasive/robotic CS and transcatheter valvular therapy with a close attention to new device/AI-incorporated tools to address valvular pathology. Edoardo is particularly interested in scientific education and the podcast and broadcast that is leading for EHJIMP and ESC are the main vectors of it.
